# Distinct roles of *GRIN2A* and *GRIN2B* variants in neurological conditions

**DOI:** 10.12688/f1000research.18949.1

**Published:** 2019-11-20

**Authors:** Scott J Myers, Hongjie Yuan, Jing-Qiong Kang, Francis Chee Kuan Tan, Stephen F Traynelis, Chian-Ming Low

**Affiliations:** 1 Center for Functional Evaluation of Rare Variants (CFERV), Emory University, Atlanta, GA, USA; 2 Department of Pharmacology and Chemical Biology, Emory University, Atlanta, GA, USA; 3 Department of Neurology, Vanderbilt Brain Institute, Vanderbilt Kennedy Center of Human Development, Vanderbilt University, Nashville, TN, USA; 4 Department of Anaesthesia, Yong Loo Lin School of Medicine, National University of Singapore, Singapore, Singapore; 5 Department of Pharmacology, Yong Loo Lin School of Medicine, National University of Singapore, Singapore, Singapore

**Keywords:** NMDA receptors, GRIN2A, GRIN2B, GluN2A, GluN2B, mutations, neurological disorder, psychiatric disorders, autism, epilepsy, intellectual disability, ADHD, schizophrenia, precision medicine

## Abstract

Rapid advances in sequencing technology have led to an explosive increase in the number of genetic variants identified in patients with neurological disease and have also enabled the assembly of a robust database of variants in healthy individuals. A surprising number of variants in the *GRIN* genes that encode *N*-methyl-D-aspartate (NMDA) glutamatergic receptor subunits have been found in patients with various neuropsychiatric disorders, including autism spectrum disorders, epilepsy, intellectual disability, attention-deficit/hyperactivity disorder, and schizophrenia. This review compares and contrasts the available information describing the clinical and functional consequences of genetic variations in *GRIN2A* and *GRIN2B.* Comparison of clinical phenotypes shows that *GRIN2A* variants are commonly associated with an epileptic phenotype but that *GRIN2B* variants are commonly found in patients with neurodevelopmental disorders. These observations emphasize the distinct roles that the gene products serve in circuit function and suggest that functional analysis of *GRIN2A* and *GRIN2B* variation may provide insight into the molecular mechanisms, which will allow more accurate subclassification of clinical phenotypes. Furthermore, characterization of the pharmacological properties of variant receptors could provide the first opportunity for translational therapeutic strategies for these *GRIN*-related neurological and psychiatric disorders.

## Introduction

Ionotropic glutamate receptors are ligand-gated ion channels that mediate excitatory synaptic transmission throughout the central nervous system. These receptors can be classified into at least three distinct families, and nomenclature is based on the initial discovery of selective activating agonists AMPA, kainate, and *N*-methyl- d-aspartate (NMDA) for their corresponding receptors, which arise from *GRIA*, *GRIK*, and *GRIN* genes, respectively. The *GRIN* gene family encodes three classes of NMDA receptor (NMDAR) subunits: the glycine-binding GluN1 (product of *GRIN1*), glutamate-binding GluN2 ( *GRIN2A*, *GRIN2B*, *GRIN2C*, and *GRIN2D*), and the enigmatic glycine-binding GluN3 ( *GRIN3A* and *GRIN3B*), the role of which remains poorly understood ^1, 2^. Most NMDARs are tetrameric assemblies of two GluN1 and two GluN2 subunits. In terms of the evolutionary history of the NMDAR, it appears that four GluN2 paralogs (GluN2A–D) were produced by two rounds of gene duplication in a common vertebrate ancestor; the rounds diverged during early vertebrate evolution principally at their carboxyl-terminal domain (CTD) ^3^. The first round of duplication gave rise to two GluN2 genes (the ancestors of GluN2A/B and GluN2C/D), and the second round gave rise to the four extant paralogs ^4^. Having a common ancestry, GluN2A and GluN2B molecular structure and function should be similar, except for the divergent CTDs. However, there are strong differences between these two subunits on almost every level, including in how clinically relevant missense variants impact the receptor and patient. In this review, we will focus on the molecular and functional basis as to why GluN2A and GluN2B show strikingly different effects when missense mutations arise (for example, *de novo* in key gating motifs and different neurological disorders).

### Glutamate receptor structure and function

**Figure 1.**
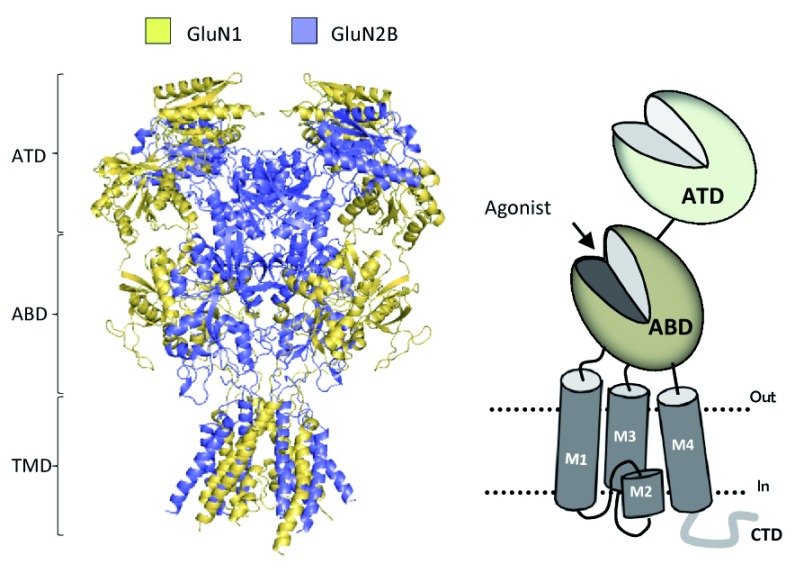
Domains of *N*-methyl- d-aspartate (NMDA) receptors. The crystal structure for GluN1/GluN2B receptors is shown in the left panel ^13^ depicting the amino-terminal domain (ATD), the agonist-binding domain (ABD), and the transmembrane domain (TMD). Not shown is the intracellular carboxyl-terminal domain (CTD). The right panel displays a schematic of a *GRIN* subunit, and the subdomains and the clamshell features of the ATD and ABD are indicated.

All NMDAR subunits contain four semi-autonomous domains: an amino-terminal domain (ATD), agonist-binding domain (ABD), transmembrane domain (TMD), and CTD ( Figure 1). The bilobed ABD of GluN2 binds l-glutamate within a cleft between two rigid lobes, S1 and S2, which form a clamshell-like structure that undergoes pronounced conformation changes upon ligand binding. The S1 lobe of the ABD, which resides distal to the ion channel, forms an interface between the ABD of adjacent subunits, allowing them to act as dimers ^5^. For NMDARs, one GluN1 and one GluN2 ABD form a dimer, two of which exist within each tetrameric receptor ^6^. The S2 lobe that is proximal to the channel contains primarily the polypeptide chain that connects the two transmembrane helices (M1 and M3) through flexible linkers and a two-turn helix (the pre-M1 helix) that lies parallel to the plane of the membrane. The S2 lobe undergoes considerable movement as the agonist binds to “close the clamshell” within the ABD of each subunit, which is the initial conformational change of several that ultimately lead to opening of the ion channel pore ^7^. This combination of agonist binding and clamshell closure provides the energy to drive channel opening in all ionotropic glutamate receptors ^6, 8– 11^.

Most information on NMDAR location and function exists for diheteromeric receptors that are a tetrameric assembly of two GluN1 subunits and two identical GluN2 subunits ^1, 2^. NMDARs are maximally activated when glycine binds to the ABD of GluN1 and l-glutamate binds to the ABD of GluN2 ^1^. Three transmembrane helices (M1, M3, and M4) form the pore and are directly coupled to the ABD in all glutamate receptor subunits, and the pore is lined by a re-entrant loop (referred to as M2) ^12, 13^ that controls ion permeation and block ^14^. Not surprisingly, single amino acid variants in these transmembrane helices, in the linkers that couple transmembrane helices to the ABD, and in the pore-lining re-entrant M2 loop can affect gating, ion permeation, and block ^15– 21^. Part of the activation gate—the structure that occludes the flux of ions in the closed state—involves the M3 segment, including a highly conserved motif (SYTANLAAF) ^10^. The process of opening and closing is highly dependent on these nine residues, residues in the pre-M1 region and a region preceding the fourth TMD ^1, 6, 22– 24^.

The NMDAR is permeable to Ca ^2+^ in addition to Na ^+^ and K ^+ 14, 22, 25– 27^, and the intraneuronal Ca ^2+^ entry subsequent to NMDAR activation can engage intracellular signaling systems that lead to changes in gene expression ^28^, changes in post-translational modifications ^29^, and ultimately changes in synaptic strength ^30^. Once the pore opens, extracellular Mg ^2+^ can join the traffic of ions moving through the channel to reach a deep binding site in the pore, the occupancy of which establishes channel block in a voltage-dependent manner (reviewed in 14). This endows the receptor with the ability to detect neuronal activity (in the form of depolarization) and simultaneous synaptic activity (in the form of release of glutamate). This coincidence detector is a central feature enabling NMDARs to participate in some, but not all, forms of synaptic plasticity ^31^. Some NMDARs can undergo desensitization during persistent activation ^32^, and the time course of desensitization for NMDARs is much slower than that for AMPA receptors ^1^. Both speed and extent of desensitization are subunit-dependent ^33, 34^, providing further separation of temporal signaling properties that depend on the frequency of synaptic input ^35^.

There are profound differences in the properties of NMDARs that contain GluN2A compared with GluN2B. For example, the open probability with GluN2A is higher than with GluN2B ^35, 36^. In addition, glutamate and glycine are both less potent at GluN2A compared with GluN2B ^37^ and thus GluN2A-containing NMDARs show a faster deactivation time course following removal of glutamate than GluN2B-containing NMDARs ^38^. The deactivation time course following glutamate removal sets the duration of the synaptic current ^39^ and thus GluN2A NMDARs will produce a faster synaptic current in comparison with GluN2B. GluN2A-containing receptors also desensitize more rapidly than GluN2B-containing receptors, which show a much slower desensitization time course ^35^. These two receptors show different sensitivity to extracellular negative allosteric modulators such as Zn ^2+ 40, 41^, and the Zn ^2+^ binding site in the ATD shows much higher potency for GluN2A than GluN2B ^42^. These functional properties, as well as the intracellular CTD that controls receptor targeting to different regions of the plasma membrane, will enable a variety of distinct functions for GluN2A- and GluN2B-containing NMDARs in neurons. There is strong evidence for perisynaptic NMDARs which could play a range of different roles ^43, 44^. Some evidence suggests that GluN2A preferentially distributes to the postsynaptic density, compared with GluN2B, which also distributes throughout the dendrite at extrasynaptic sites ^45^. This distinct localization has also been suggested to influence the participation of these two receptor subunits in different forms of synaptic plasticity ^46– 48^. Moreover, there are distinct roles of NMDARs of different subunit composition in neuroprotective signaling and cell death signaling that reflect both their localization and ability to pass current and Ca ^2+ 49– 52^. However, the subcellular distributions of GluN2A and GluN2B are not absolute, and both subunits can be found both synaptically and extrasynaptically.

### Developmental expression profile of GluN2A and GluN2B

The temporal expression profile of different NMDAR subunits is precisely controlled to coincide with critical periods in the development of different brain structures ^53– 58^. Indeed, *GRIN2* gene expression in the brain changes throughout the postnatal developmental stages ^59^. The GluN2B subunit is highly expressed in the prenatal stages and its expression drops at the postnatal stages, becoming focally expressed in the forebrain. However, GluN2A is expressed at apparently low levels in the prenatal stages and increases upon birth ^53, 60^. In rodents, *GRIN2A* mRNA appears detectable by *in situ* hybridization studies around postnatal day 6. There is a progressive developmental change from predominantly GluN1/GluN2B receptors to GluN1/GluN2A receptors in many brain regions ^58, 61– 63^, including thalamic and cortical neurons during the early postnatal development ^64^. The decrease in GluN2B-containing NMDARs at synapses is corroborated with the detection of shorter excitatory postsynaptic currents and a decrease in sensitivity to a specific GluN1/GluN2B receptor antagonist, ifenprodil ^65, 66^. The prenatal expression of *GRIN2B* in NMDAR subunit has been taken as evidence for an important role in brain development, circuit formation, and possibly cell migration and differentiation ^67^. In early postnatal development and late embryogenesis, GluN2B expression dominates during rapid cortical synaptogenesis ^68^. Kutsuwada *et al*. observed neonate lethality of global *GRIN2B* knockout mice ^69^, whereas Tang *et al*. reported that overexpression of *GRIN2B* in the forebrain of mice enhanced spatial memory performance and long-term hippocampal potentiation ^70^. ****

It is not surprising that the many recent reports on human variants in *GRIN* genes show different clinical phenotypes (discussed below) given that the modular structure of the receptor can compartmentalize the different actions of variants in different domains, thereby impacting different functional modalities. In addition, the distinct roles of GluN2A and GluN2B in synaptic signaling and circuit function enabled by their different developmental expression profile ^58, 61– 63^, the compartmentalization ^62^, and the functional attributes mean that variants in these two genes could have very different effects and different age-dependent phenotypes. For example, more pronounced effects might be observed for *GRIN2B* variants in terms of neurodevelopment, and effects of the mutations might present early in postnatal stages and manifest as neurodevelopmental disorders, developmental delays (DDs), and intellectual disability (ID) ^71^. However, the effect of *GRIN2A* will start to show in the later postnatal stage as the expression of *GRIN2A* starts to increase and thus the influence of GluN2A-containing NMDARs becomes important. Most of the GluN2A variants were identified in patients with epileptic seizures and epileptic encephalopathies ^72^. In most cases when variants reduce Mg ^2+^ inhibition and otherwise show enhanced NMDAR function, the patients show epileptic encephalopathy (EE), which may reflect not just DD from persistent and intractable seizures but perhaps excitotoxic neuronal cell death ^73^. Interestingly, variants in GluN1 will impact all NMDARs and thus would be expected to have even further distinct effects compared with *GRIN2A* and *GRIN2B* ^74^. However, in this review, we restrict our summary of recent studies to rare *de novo* variants discovered in the *GRIN2A* and *GRIN2B* genes. We highlight an emerging understanding of the functional and clinical consequences of these variants in the context of receptor expression, localization, and the unique roles that GluN2A and GluN2B subunits play. We speculate that viewing the phenotypic differences for patients with *GRIN2A* and *GRIN2B* through the lenses of these different properties will provide greater insight into disease mechanism and this information will create the possibility of instituting mechanism-based novel therapeutic treatments.

### The increase in genetic information identifies a large number of disease-associated variants

The number of rare variants associated with neurological disease is expanding rapidly. Since the identification of the first disease-causing variants in NMDARs in 2010 ^75, 76^, over 500 variants in all *GRIN* genes coding for NMDARs—found in all four semi-autonomous domains, (the ATD, the ABD, the TMD, and the CTD)—have been reported in ClinVar or from patient cohorts in the literature. These include 249 variants in the *GRIN2A* and 204 variants in the *GRIN2B* (ClinVar). The increasing use of next-generation whole exome sequencing in clinical practice promises to identify even more rare *de novo* variants in *GRIN* genes linked to neurological disorders as diagnostic whole exome sequencing efforts expand and become common practice outside academic medical centers ^71, 77, 78^. The rapid identification of numerous rare genetic variants should be followed by functional analysis of these variants, which, though more time-intensive, is an essential step in establishing their role in neurological disease. In addition, functional evaluation provides mechanistic insight toward disease etiology and potential treatment options. Fortunately, the NMDARs encoded by these genes can be easily expressed in heterologous systems and their function, though complex, is reasonably well understood ^1, 2, 6^. Multiple groups are expanding efforts to fill the gap between genetic identification of rare variants and elucidation of their functional consequences ^6, 23, 73, 74, 79– 98^. With continued effort, all disease-associated variants eventually should be identified and functional characterization of these variants will inform the subdivision of variants into a limited set of groups with more homogenous clinical and functional phenotypes. This will allow direct comparison of variants with similar effects between different subunits and help elucidate the roles that these subunits play during development. It will also transform diagnoses and treatment options since variant function eventually will be readily available for clinicians in real time. However, at the moment, there remains a significant lag in efforts to obtain this functional information compared with the amount of new sequencing being performed.

## Comparison of patient phenotype for *GRIN2A* and *GRIN2B* missense and nonsense variants

Among the variants identified in the *GRIN* gene family, those in *GRIN2A* (46%) and *GRIN2B* (38%) account for the vast majority, followed by *GRIN1* variants (14%; ClinVar) ^72^. It is important to note that all of these genes can co-assemble to form functional receptors, meaning that the *GRIN* variants should be thought of as a larger set of variants since variants in all three genes can produce similar gain-of-function (GoF) or loss-of-function (LoF) effects on NMDARs. Moreover, every NMDAR contains GluN1 and thus these variants in particular will impact both GluN2B- and GluN2A-containing NMDARs. However, there will be differences in the overall effects for *GRIN2A* versus *GRIN2B* genes depending on their regional and developmental expression profile, in addition to the different roles that they can play in circuit function. Thus, it will be useful to stratify variants by gene and by GoF and LoF even though they impact an overlapping set of NMDAR complexes expressed in the brain. Given that the most common *GRIN* variants are in *GRIN2A* and *GRIN2B*, a comprehensive evaluation of these two subunits provides an opportunity to understand the structural, functional, and genetic bases for disorders that these patients have. Functional consequences of many *GRIN2A* and *GRIN2B* variants have been assessed in heterologous expression systems and so we will focus on the effects of rare variants in these two genes.

An assessment of the genetic variation in the healthy population together with an evaluation of *GRIN2A* and *GRIN2B* variants in patients with neurological disease provides information about the regional tolerance of different domains of the GluN2 subunit. This approach reveals insight into protein function and information about the regions of the receptor that cannot tolerate even modest changes in amino acid side chain properties ^81^. Such regions appear to have undergone purifying selection and this determination can aid in future *in silico* predictions of the impact of missense variants. Evaluation of *GRIN2A* and *GRIN2B* revealed that the ABD, TMDs, and the linker regions between these domains were particularly intolerant to genetic variation and suggests that these domains are under greater selection pressure ^82, 83^. These two regions appear to harbor the most disease-associated variants within the *GRIN2A* and *GRIN2B* genes ^72, 82– 84^. Evaluation of phenotypic severity for a cohort of patients harboring *GRIN2A* variants showed stratification in severity across variants with different functional effects and localizations ^85^. There are some subtle differences in regions that are insensitive to variation that reflects different functions of GluN2A and GluN2B subunits. There are examples where different patients (that is, different genetic backgrounds) harbor the same *de novo GRIN* missense variant in their genome; in these cases, the patients display similar but non-identical clinical phenotypes ^21, 23, 84– 88^. As the databases of genetic variation within the standing population expand, there will be an ever-increasing precision with which we can define intolerant regions, and we expect that more specific examples of intolerant regions that differ between the two subunits will emerge. In this review, we also compiled a list of *GRIN2A* and *GRIN2B* variants with phenotypes published in the literature and not found in the gnomAD database ( http://gnomad.broadinstitute.org; Table 1).

**Table 1.** Phenotypes reported across *GRIN2A* and *GRIN2B* subdomains.

Phenotypes (top) or Variant Type (bottom)	# reported in *GRIN2A* / # reported in *GRIN2B*					
	ATD	ABD	ABD-TMD Linkers	TMD	CTD	Other
Epi	25 / 3	41 / 13	5 / 4	19 / 12	9 / 0	26 / 1
ID	28 / 13	32 / 35	4 / 9	19 / 23	8 / 9	22 / 15
ASD	4 / 6	5 / 13		4 / 3	2 / 6	1 / 5
Language delay/Verbal dyspraxia/Aphasia syndrome	14 / 0	22 / 0	5 / 1	10 / 0	1 / 1	7 / 0
LKS	1 / 0	3 / 0	1 / 0		1 / 0	1 / 0
ADHD/Rett-like syndrome/Behaviorial anomalies	2 / 1	3 / 0	1 / 1	1 / 1		1 / 2
IS	1 / 0	1 / 0		0 / 2		
CVI				1 / 3		
Hypotonia/Dystonia	3 / 0	0 / 1	0 / 1	9 / 1		2 / 1
LGS		0 / 1				
MD		3 / 1	3 / 1	4 / 2		0 / 1
West syndrome		0 / 1		0 / 2		
Dysmorphic features	1 / 0	1 / 0	0 / 1	2 / 1		3 / 0
SCZ/Bipolar disorder	1 / 2				1 / 2	2 / 0
Macrocephaly or Abnormality of nervous system					1 / 1	3 / 1
Missense variant	12 / 7	31 / 29	4 / 8	20 / 23	7 / 8	
Nonsense variant	8 / 4	3 / 2	0 / 1	0 / 2	3 / 2	
Splice junction variant	9 / 0	7 / 3	1 / 0	0 / 1		
Frame-shift variant	5 / 4	9 / 1		4 / 0	2 / 3	
Indel variant			1 / 0		1 / 1	
Other						31 / 17

The table compares genetic variants in *GRIN2A* and *GRIN2B* genes with phenotypes as reported in the literature and absent in the gnomAD database ( http://gnomad.broadinstitute.org). ABD, agonist-binding domain; ADHD, attention-deficit/hyperactivity disorder; ASD, autism spectrum disorder; ATD, amino-terminal domain; CTD, carboxyl-terminal domain; CVI, cerebral visual impairment; Epi, epilepsy/seizures; ID, intellectual disability (including developmental delay); IS, infantile spasms; LGS, Lennox–Gastaut syndrome; LKS, Landau–Kleffner syndrome; MD, movement disorder; SCZ, schizophrenia; and TMD, transmembrane domains (M1-M4). Other, refers to chromosome deletions, insertions, duplications that affect *GRIN2A* or *GRIN2B* genes. References 21, 73, 75, 76, 80, 82– 86, 89– 92, 94, 96, 98– 101, 103– 153.

### GRIN2A predominantly is associated with epilepsy and intellectual disability

More than 240 missense and nonsense variants have been reported for *GRIN2A*. *De novo* variants in *GRIN2A* can be found in phenotypically normal neonates with a structurally normal brain at birth ^85, 89^. Multiple patients appear to have had uncomplicated pregnancies and normal deliveries with excellent appearance, pulse, grimace, activity, respiration (APGAR) scores and no immediate complications. However, patients can begin to show neurological abnormalities at a young age (during the first year of life) ^89^, presumably as a result of increasing expression of *GRIN2A* with development ^60^. This most often manifests as abnormal electroencephalography (EEG) and myoclonic jerks progressing to a seizure disorder. Several studies have suggested that benign focal epilepsy with centrotemporal spikes (BECTS) seems to be caused by both missense and nonsense *de novo* mutations within the *GRIN2A* gene: Three reports from 2013 showed that *GRIN2A* gene variants are more likely to occur in epilepsy subtypes that are believed to be a more severe variant of BECTS such as atypical benign partial epilepsy of childhood, Landau–Kleffner syndrome, and continuous spike waves during slow wave sleep ^99– 101^. An intriguing aspect of these epilepsy patients who harbor *GRIN2A* variants is that the variants can produce both GoF and LoF, as inferred by nonsense variants that produce protein truncation. Patients with a deletion that removes the *GRIN2A* gene also show hyperexcitability ^76^. The mechanisms that ultimately promote hyperexcitability in patients lacking *GRIN2A* are not yet known, but likely due to haploinsufficiency although it appears that they in some way enhance circuit excitability ^102^. Interestingly, there is no firm evidence to suggest that *GRIN2A* variants contribute to the two most common epilepsy syndromes: idiopathic generalized epilepsy and temporal lobe epilepsy ^154^. *GRIN2A* variants are linked to autism spectrum disorder (ASD) but to a lesser degree than seen with *GRIN2B* variants ( Table 1).

*GRIN* variants have been identified both by screening of a select set of genes assembled as a panel or by whole exome sequencing, which provides good coverage over much of the exome, although certain GC-rich regions of DNA (for example, the 5' region of *GRIN2D*) are often under-represented. Nevertheless, these approaches have discovered a large number of *de novo* variants in neurological patients. A functional analysis has been published in the peer-reviewed literature for a number of *GRIN2A* variants ^73, 75, 79, 83, 86, 90– 93^. In addition, there is a comprehensive functional summary on the websites as a resource ( http://functionalvariants.emory.edu). Below, a subset of these published variants is discussed to illustrate some important commonalities and distinctions.

The functional consequences of several variants reported in the gnomAD database, some of which showed functional changes, were evaluated. One clinical case report is for a missense variant (GluN2A-V452M) from a patient with early infantile EE/Ohtahara syndrome ^155^. Another example of a variant proposed to cause a disease phenotype is a heterozygous *GRIN2A* variant that produces GluN2A-N447K in a male with Rolandic epilepsy. EEG monitoring showed remarkable interictal high voltage spikes and spike-and-slow waves in the bilateral central-temporal regions, predominantly on the right hemisphere. The GluN2A-N447K variant is located in the S1 segment of the extracellular ABD of GluN2A. Residue N447 is highly conserved across higher vertebrates yet the Asn447Lys variant is present multiple times in gnomAD. Whole cell patch clamp recording of GluN2A-N447K reveals a GoF effect and an increase in NMDAR current density by about 1.2-fold, an enhancement of glutamate potency by two fold, and reduced sensitivity to Mg ^2+^ inhibition ^93^. Experimental substitution of Asn447 to alanine (uncharged) or glutamic acid (negatively charged) did not change NMDAR function, suggesting that the positive charge associated with lysine may have altered NMDAR function. The patient became seizure-free when treated with a combination of valproate and lamotrigine ^93^.

A child with epileptic encephalopathy (EE) and severe cognitive impairment possessed a GoF missense *GRIN2A* variant that produced GluN2A-L812M. This position in the linker between the ABD and TMD regions is intolerant to change, as all amino acid substitutions (seven to date) at this position produced a GoF variant that showed increased agonist potency, increased open probability, and reduced sensitivity to endogenous negative modulators such as extracellular Mg ^2+^ and protons ^73^. The variant NMDARs’ activities were enhanced by virtually every measure and would be expected to lead to profound overactivation, which could drive excitotoxic mechanisms. Memantine is able to inhibit the GluN2A-L812M-containing NMDARs, and treatment with memantine led to a persistent reduction of the child’s seizure burden ^89^.

A heterologous *de novo* variant was found in a 3-year-old female with early-onset EE, abnormal EEG, and severe DD ^75^. The variant substituted an evolutionarily conserved asparagine for a lysine (GluN2A-N615K) in the membrane re-entrant loop, which lines the channel pore and creates a constriction that controls ion selectivity of the channel ^6, 156, 157^. This variant alters the voltage-dependent channel block by Mg ^2+^ and decreases in Ca ^2+^ permeability. Co-expression of GluN2-N615K with GluN1 and wild-type GluN2A in the same receptor complex, called triheteromeric receptors (comprised of GluN1:GluN2A:GluN1:GluN2A-N615K subunits), produces an intermediate effect, indicating that the negative impact caused by the variant on channel properties cannot be fully negated by the presence of one normal subunit copy of GluN2A in the receptor complex ^21, 92^.

### *GRIN2B* is predominantly associated with developmental delay, intellectual disability, and autism spectrum disorder

Over 200 variants in *GRIN2B* are found in patients from cohorts with any one of several neurodevelopmental disorders ^71^ such as ID (including DD) ^14, 75, 80, 82– 84, 91, 94– 96, 98, 103– 108, 111, 112, 114, 120, 122, 123, 128, 130, 135, 139– 147, 149– 153^, ASD ^75, 80, 82, 84, 91, 98, 104, 105, 108, 111, 135, 140, 144, 148, 151– 153^, EE and seizure disorders ^21, 80, 82, 84, 98, 103, 120, 130, 135, 138, 139, 141, 145^, schizophrenia ^71, 111, 114, 137, 148^, and, to a lesser extent, attention-deficit/hyperactivity disorder ^80, 84, 106^, cerebral visual impairment ^158, 146^, and Alzheimer’s disease ^159^ have been reported in the literature. For these various phenotypes, virtually all of the patients display mild to profound DD or ID or both. In addition to exhibiting these neurological phenotypes, some patients exhibit abnormalities in muscle tone that includes spasticity or hypotonia ^158, 151^. *GRIN2B* has been linked as a potential gene in which variations could increase the risk of autism ^104, 105, 160^. A number of other characteristics, including microcephaly, movement disorders, Rett-like syndrome, language disorders have been observed in some patients ^75, 96, 115, 142, 158^.

The *GRIN2B* variants identified thus far occur throughout the entire NMDAR subunit protein. That is, missense and nonsense variants have been identified in the ATD, ABD, TMD, and CTD domains. Homozygous *Grin2b*-deletion mice die at early postnatal stages because of impaired suckling response and show impaired hippocampal long-term depression, whereas heterozygous mice show reduced expression of GluN2B but survive ^69^. Thus, *GRIN2B* is an essential gene for normal development. *GRIN2B de novo* variants with neurological diseases have been reviewed ^71, 77, 161^, and functional data exist for many *GRIN2B* variants in published scientific journals (see below) or online databases ( http://functionalvariants.emory.edu).

Large cohort studies for ID or ASD have identified that LoF variants in *GRIN2B* segregate with a broad spectrum of these neurological phenotypes ^75, 105^. One such LoF variant is the missense variant GluN2B-E413G ^106^, which produces DD and ID. Studies conducted on neural progenitor cells (NPCs) generated from induced pluripotent stem cells found that neurons with heterozygous GluN2B-E413G, which is in the glutamate-binding pocket, caused a 50-fold decrease in glutamate signaling and reduced the maturation states of the neurons. Verification of failure to phosphorylate serine 133 in cAMP response element-binding protein (CREB) by NMDAR stimulation further confirms that the E413G variant in GluN2B impaired NMDAR signaling in the NPCs ^94^. This study shows that GluN2B-containing NMDARs are critical for signal transduction in neural stem cells and deﬁcits in this process impair cellular differentiation. The E413G variant is in close proximity to the glutamate-binding site but is not in physical contact with the agonist glutamate. Modelling of the protein structure suggests that GluN2B-E413 can alter agonist dissociation by increasing the ability of water to compete with agonist binding, thereby accelerating glutamate unbinding and likely rendering the synaptic NMDAR response time course briefer than that for wild-type NMDARs ^82, 95^.

The *GRIN2B* variant encoding GluN2B-C461F, which was identified in a patient with Lennox–Gastaut syndrome and autistic features, is a LoF NMDAR variant. Cys461 is located in S1 of the ABD, close to the orthosteric glutamate-binding site. When co-transfected with GluN1-4b, an early developmental isoform of *GRIN1*, GluN1:GluN2B-C461F receptors reduced glutamate potency by 71-fold compared with wild-type controls ^80^. This is in keeping with decreased glutamatergic neurotransmission in animal models of ASD (for example, BTBR mice), in which the phenotype can be improved by a selective AMPAKINE (AMPA receptor–positive allosteric modulator). Lennox–Gastaut syndrome is a severe type of childhood epilepsy. Also, during early development, high expression of variant GluN2B subunits could compromise neurotransmitter-based signaling (for example, GABA release via presynaptic NMDARs), circuit operation between distinct cell types (interneurons/principal neurons), and the balance of excitation and inhibition ^80^. This is in line with the association of mutations in *GABRB3* with Lennox–Gastaut syndrome and autism ^162– 164^. In addition, synaptic pruning and synapse refinement are GluN2-dependent events that occur generally at a time of a switch in subunit expression from GluN2B to GluN2A ^64^ and thus could be influenced by altered GluN2B function. This could be one contributing factor that is an underlying substrate for developmental effects of *GRIN2B* variants ^165^.

The GluN2B-P553L variant was identified in a patient with severe ID ^107^. This variant was found to minimally affect glutamate potency, but the rate of desensitization of GluN1–GluN2B-P553L was markedly increased and currents were small in HEK cells ^80, 83^. Pro553 is located at the proximal end of the first TMD, within the pre-M1 linker that connects S1 to M1. Spatially, P553 is adjacent to the highly conserved nine-residue signal-transduction element (SYTANLAAF) in M3 of the same subunit, which is involved in coupling ligand binding to channel opening, and controls channel open probability ^6^. Thus, the variant Pro553Leu may form different interactions with Asn649 or Leu650 (or both) in the SYTANLAAF motif, which could interfere with gating.

GluN2B-N615I and GluN2B-V618G variants are both associated with West syndrome, which is a triad of infantile spasms, hypsarrhythmia, and ID ^103^. GluN2B-N615 and -V618 are located in the M2 re-entrant loop, which forms part of the ion channel. N615 is located just above the narrowest constriction in the pore, which is also influenced by analogous residues in the GluN1 subunit (for example, GluN1-N616). Val618 in GluN2B is located deep in the channel pore, within the M2-M3 linker and with the CH _3_ side chain that has been suggested to be rotated away from the channel pore. This side-chain will interact with residues in M2 and M3 membrane helices of GluN1 ^80, 98^. For GluN2B-N615I and GluN2B-V618G, voltage-dependent Mg ^2+^ inhibition was lost, resulting in a GoF phenotype that will allow increased NMDAR current under normal resting conditions, which may underlie increased neuronal excitability in West syndrome. The onset of symptoms in the patient coincided with the high expression profile of GluN2B in late infancy (<1 year old) ^62, 103^.

### Variants at the same residue position in GluN2A and GluN2B resulted in different disease phenotypes

A variant in both *GRIN2A* and *GRIN2B* that occurs at the same homologous position of these GluN2 subunits in NMDAR has been identified. Functionally, both GluN2A-N615K and GluN2B-N615I and GluN2B-N615K variants that substitute an evolutionarily conserved asparagine in the membrane re-entrant loop resulted in a loss of Mg ^2+^ block ^21, 75, 80, 84^. However, the resulting neurological phenotypes were found to be different. A 3-year-old female with a GluN2A-N615K variant exhibits early-onset EE, an abnormal EEG, and a severe DD ^75^, whereas for a GluN2B-N615I variant, the patient had West syndrome, hypsarrhythmia, and ID due to neurodevelopment disorders ^80, 84^ and GluN2B-N615K patient had ID and DD ^84^. This further validates our hypothesis that rare variants in intolerant domains in *GRIN2A* are more likely to cause an epileptic phenotype but that variants in *GRIN2B* are more aligned with abnormal developmental phenotypes.

Other cases for which the genetic variants were found on different GluN2 subunits at a homologous amino acid are GluN2A-P552R and GluN2B-P553L. GluN2A-P552R was identified in a patient with delayed psycho-motor development, ID, inability to speak, and epilepsy since 9 months of age ^83, 107^. The GluN2A-P552R variant shows increased sensitivity to glutamate and glycine but with a slower activation and deactivation time course. The GluN2B-P553L variant presents in a patient with severe ID and DD ^80, 83, 98, 107^. This variant was found to reduce glutamate potency by 1.7-fold but increase the rate of glutamate current desensitization ^80, 83^. Thus, whereas one variant should increase charge transfer for each synaptic current, the other should diminish it.

In most situations, the GluN2A or GluN2B variants exist as a single allele (that is, are heterozygous) in the patients and therefore patients also have one copy of the wild-type allele. Therefore, it would be important to understand the effects of rare variants in both diheteromeric and triheteromeric NMDARs. As the NMDARs exist as a heterotetramer with two obligatory GluN1 subunits, the possibility exists that a diheteromer (for example, GluN1/GluN2A/GluN1/GluN2A) or triheteromer (for example, GluN1/GluN2A/GluN1/GluN2B) harbors a single disease variant GluN2 subunit.

To understand the *GRIN2* variant effects in the presence of wild-type allele, NMDAR subunits were engineered to co-express as GluN1/GluN2A/GluN1/GluN2A-P552R and GluN1/GluN2A-P552R/GluN1/GluN2A-P552R, analyzed for response time course to glutamate and glycine, and compared with wild-type control GluN1/GluN2A. Single channel recording from single copy variant–containing receptors did not alter mean channel open time or chord conductance as compared with the wild-type channel. For the NMDARs with two copies of GluN2A-P552R, there is a significant increase in mean open time and reduced channel conductance when compared with the wild-type or single copy mutant. These data suggest that the GluN2A-P552R variants can alter stability and conformation of the open pore or its access portals only when both GluN2A subunits contain the P552R variant ^83^.

## Precision medicine

As more precise diagnoses for individuals are achieved, the basis of disease etiology will be better defined. We anticipate that novel drug development and a more mechanism-based use of currently approved drugs can improve clinical outcomes. For pharmacological treatment, the knowledge of risk factors, disease subtype, or underlying genetic variation should allow a choice of therapies proven effective in individuals with similar characteristics. For rare variants that are thought to contribute to or cause a disease, unique treatments that alter the function of the target or its downstream effects could provide novel therapies. This collection of ideas together can be described as precision medicine, an idea that is enabled by recent advances in technology on multiple fronts. There are several opportunities for potential precision medicine among the *GRIN* variants. For example, it seems reasonable that therapies already approved by the US Food and Drug Administration that inhibit NMDAR receptors might have utility against symptoms produced by GoF *GRIN* variants provided that some of the ongoing neurological symptoms reflect expression of aberrant protein rather than errant processes during development or cell loss driven by excitotoxicity. Likewise, although there are no currently available NMDAR potentiators approved for clinical use, supplementation with the co-agonists glycine, d-serine, or perhaps d-cycloserine might provide a way to augment NMDAR function, although there remains no systematic evaluation of this possibility in animal models or patients ^96^.

Personalized medicine through pharmacological intervention on patients harboring *de novo GRIN2A* and *GRIN2B* variants has been attempted. However, caution must be exercised as the potential drugs available (for example, memantine) are non-selective blockers of NMDARs, meaning that one may induce a block at some sites that are not contributing to the pathology. Memantine binding was also affected by the presence of bound Mg ^2+^ in the channel, which reduced memantine potency more for GluN2A and GluN2B than GluN2C or GluN2D ^166^. Kinetic and molecular docking results indicated overlapping sites for Mg ^2+^ and memantine, with Mg ^2+^ binding at the level of the asparagine residues, whereas memantine binds just above the channel pore ^167^. Among the NMDAR subtypes, memantine has been suggested to be more potent at GluN2C- and GluN2D-containing NMDARs, the latter of which are expressed in GABAergic interneurons ^53, 168^. Nevertheless, over-active receptors may be attenuated, and some degree of voltage-dependent block potentially restored, to compensate for a reduced Mg ^2+^ block by a rare variant. However, again caution is emphasized as several distinct disease variants in the channel pore can also alter the effect of candidate therapies, and already there are examples among the *GRIN* genes in which the variant renders a potential drug candidate less effective. For example, memantine was more potent at GluN1/GluN2B-N615I and less potent at GluN1/GluN2B-V618G, compared with wild-type receptors, which holds important implications for therapeutics. Interestingly, dextromethorphan showed increased potency for some variant receptors compared with wild-type receptors ^90^.

Of the various mutations studied in detail, two result in LoF (C461F and P553L) and two in GoF (N615I and V618G). On this basis, memantine cannot be considered an all-encompassing treatment for NMDAR mutations and will be therapeutically beneficial for only selected GoF channel variants unless compensatory NMDAR is large. Still, there are some examples in which NMDAR block by memantine showed some utility ^86, 89, 97^. The inhibition of NMDAR-mediated currents by memantine at negative membrane potentials was comparable between wild-type and N615I- or V618G-expressing neurons. This supports a role for this medication as a potential therapy to mimic a loss Mg ^2+^ block at the potentials in neurons.

## References

[1] Traynelis SF Wollmuth LP McBain CJ: Glutamate receptor ion channels: structure, regulation, and function. *Pharmacol Rev.* 2010;62(3):405–96. 10.1124/pr.109.002451 20716669 PMC2964903

[2] Paoletti P Bellone C Zhou Q: NMDA receptor subunit diversity: impact on receptor properties, synaptic plasticity and disease. *Nat Rev Neurosci.* 2013;14(6):383–400. 10.1038/nrn3504 23686171

[3] Ryan TJ Emes RD Grant SG: Evolution of NMDA receptor cytoplasmic interaction domains: implications for organisation of synaptic signalling complexes. *BMC Neurosci.* 2008;9:6. 10.1186/1471-2202-9-6 18197970 PMC2257970

[4] Ryan TJ Kopanitsa MV Indersmitten T: Evolution of GluN2A/B cytoplasmic domains diversified vertebrate synaptic plasticity and behavior. *Nat Neurosci.* 2013;16(1):25–32. 10.1038/nn.3277 23201971 PMC3979286

[5] Wang JX Furukawa H: Dissecting diverse functions of NMDA receptors by structural biology. *Curr Opin Struct Biol.* 2019;54:34–42. 10.1016/j.sbi.2018.12.009 30703613 PMC6592722

[6] Hansen KB Yi F Perszyk RE: Structure, function, and allosteric modulation of NMDA receptors. *J Gen Physiol.* 2018;150(8):1081–105. 10.1085/jgp.201812032 30037851 PMC6080888

[7] Karakas E Regan MC Furukawa H: Emerging structural insights into the function of ionotropic glutamate receptors. *Trends Biochem Sci.* 2015;40(6):328–37. 10.1016/j.tibs.2015.04.002 25941168 PMC4464829

[8] Armstrong N Sun Y Chen GQ: Structure of a glutamate-receptor ligand-binding core in complex with kainate. *Nature.* 1998;395(6705):913–7. 10.1038/27692 9804426

[9] Armstrong N Gouaux E: Mechanisms for activation and antagonism of an AMPA-sensitive glutamate receptor: crystal structures of the GluR2 ligand binding core. *Neuron.* 2000;28(1):165–81. 10.1016/s0896-6273(00)00094-5 11086992

[10] Sobolevsky AI Rosconi MP Gouaux E: X-ray structure, symmetry and mechanism of an AMPA-subtype glutamate receptor. *Nature.* 2009;462(7274):745–56. 10.1038/nature08624 19946266 PMC2861655

[11] Yelshanskaya MV Mesbahi-Vasey S Kurnikova MG: Role of the Ion Channel Extracellular Collar in AMPA Receptor Gating. *Sci Rep.* 2017;7(1):1050. 10.1038/s41598-017-01146-z 28432359 PMC5430913

[12] Lee CH Lü W Michel JC: NMDA receptor structures reveal subunit arrangement and pore architecture. *Nature.* 2014;511(7508):191–7. 10.1038/nature13548 25008524 PMC4263351

[13] Karakas E Furukawa H: Crystal structure of a heterotetrameric NMDA receptor ion channel. *Science.* 2014;344(6187):992–7. 10.1126/science.1251915 24876489 PMC4113085

[14] Glasgow NG Siegler Retchless B Johnson JW: Molecular bases of NMDA receptor subtype-dependent properties. *J Physiol.* 2015;593(1):83–95. 10.1113/jphysiol.2014.273763 25556790 PMC4293056

[15] Wollmuth LP Kuner T Seeburg PH: Differential contribution of the NR1- and NR2A-subunits to the selectivity filter of recombinant NMDA receptor channels. *J Physiol.* 1996;491(Pt 3):779–97. 10.1113/jphysiol.1996.sp021257 8815211 PMC1158818

[16] Schneggenburger R Ascher P: Coupling of permeation and gating in an NMDA-channel pore mutant. *Neuron.* 1997;18(1):167–77. 10.1016/s0896-6273(01)80055-6 9010214

[17] Zuo J de Jager PL Takahashi KA: Neurodegeneration in Lurcher mice caused by mutation in delta2 glutamate receptor gene. *Nature.* 1997;388(6644):769–73. 10.1038/42009 9285588

[18] Krupp JJ Vissel B Heinemann SF: N-terminal domains in the NR2 subunit control desensitization of NMDA receptors. *Neuron.* 1998;20(2):317–27. 10.1016/S0896-6273(00)80459-6 9491992

[19] Villarroel A Regalado MP Lerma J: Glycine-independent NMDA receptor desensitization: localization of structural determinants. *Neuron.* 1998;20(2):329–39. 10.1016/S0896-6273(00)80460-2 9491993

[20] Ren H Honse Y Karp BJ: A site in the fourth membrane-associated domain of the *N*-methyl-D-aspartate receptor regulates desensitization and ion channel gating. *J Biol Chem.* 2003;278(1):276–83. 10.1074/jbc.M209486200 12414797

[21] Li J Zhang J Tang W: *De novo GRIN* variants in NMDA receptor M2 channel pore-forming loop are associated with neurological diseases. *Hum Mutat.* 2019; in press 10.1002/humu.23895 31429998 PMC6874887

[22] Dingledine R Borges K Bowie D: The glutamate receptor ion channels. *Pharmacol Rev.* 1999;51(1):7–61. 10049997

[23] Chen W Shieh C Swanger SA: *GRIN1* mutation associated with intellectual disability alters NMDA receptor trafficking and function. *J Hum Genet.* 2017;62(6):589–97. 10.1038/jhg.2017.19 28228639 PMC5637523

[24] Gibb AJ Ogden KK McDaniel MJ: A structurally derived model of subunit-dependent NMDA receptor function. *J Physiol.* 2018;596(17):4057–89. 10.1113/JP276093 29917241 PMC6117563

[25] MacDermott AB Mayer ML Westbrook GL: NMDA-receptor activation increases cytoplasmic calcium concentration in cultured spinal cord neurones. *Nature.* 1986;321(6069):519–22. 10.1038/321519a0 3012362

[26] Schneggenburger R: Simultaneous measurement of Ca ^2+^ influx and reversal potentials in recombinant *N*-methyl-D-aspartate receptor channels. *Biophys J.* 1996;70(5):2165–74. 10.1016/S0006-3495(96)79782-5 9172740 PMC1225191

[27] Watanabe J Beck C Kuner T: DRPEER: a motif in the extracellular vestibule conferring high Ca ^2+^ flux rates in NMDA receptor channels. *J Neurosci.* 2002;22(23):10209–16. 10.1523/JNEUROSCI.22-23-10209.2002 12451122 PMC6758750

[28] Deisseroth K Heist EK Tsien RW: Translocation of calmodulin to the nucleus supports CREB phosphorylation in hippocampal neurons. *Nature.* 1998;392(6672):198–202. 10.1038/32448 9515967

[29] Bayer KU de Koninck P Leonard AS: Interaction with the NMDA receptor locks CaMKII in an active conformation. *Nature.* 2001;411(6839):801–5. 10.1038/35081080 11459059

[30] Herring BE Nicoll RA: Long-Term Potentiation: From CaMKII to AMPA Receptor Trafficking. *Annu Rev Physiol.* 2016;78:351–65. 10.1146/annurev-physiol-021014-071753 26863325

[31] Bender VA Bender KJ Brasier DJ: Two coincidence detectors for spike timing-dependent plasticity in somatosensory cortex. *J Neurosci.* 2006;26(16):4166–77. 10.1523/JNEUROSCI.0176-06.2006 16624937 PMC3071735

[32] Sather W Dieudonné S MacDonald JF: Activation and desensitization of *N*-methyl-D-aspartate receptors in nucleated outside-out patches from mouse neurones. *J Physiol.* 1992;450(1):643–72. 10.1113/jphysiol.1992.sp019148 1359126 PMC1176143

[33] Medina I Filippova N Charton G: Calcium-dependent inactivation of heteromeric NMDA receptor-channels expressed in human embryonic kidney cells. *J Physiol.* 1995;482(Pt 3):567–73. 10.1113/jphysiol.1995.sp020540 7537819 PMC1157782

[34] Price CJ Rintoul GL Baimbridge KG: Inhibition of calcium-dependent NMDA receptor current rundown by calbindin-D _28k_. *J Neurochem.* 1999;72(2):634–42. 10.1046/j.1471-4159.1999.0720634.x 9930735

[35] Erreger K Dravid SM Banke TG: Subunit-specific gating controls rat NR1/NR2A and NR1/NR2B NMDA channel kinetics and synaptic signalling profiles. *J Physiol.* 2005;563(Pt 2):345–58. 10.1113/jphysiol.2004.080028 15649985 PMC1665591

[36] Chen N Luo T Raymond LA: Subtype-dependence of NMDA receptor channel open probability. *J Neurosci.* 1999;19(16):6844–54. 10.1523/JNEUROSCI.19-16-06844.1999 10436042 PMC6782868

[37] Erreger K Geballe MT Kristensen A: Subunit-specific agonist activity at NR2A-, NR2B-, NR2C-, and NR2D-containing *N*-methyl-D-aspartate glutamate receptors. *Mol Pharmacol.* 2007;72(4):907–20. 10.1124/mol.107.037333 17622578

[38] Vicini S Wang JF Li JH: Functional and pharmacological differences between recombinant *N*-methyl-D-aspartate receptors. *J Neurophysiol.* 1998;79(2):555–66. 10.1152/jn.1998.79.2.555 9463421

[39] Lester RA Clements JD Westbrook GL: Channel kinetics determine the time course of NMDA receptor-mediated synaptic currents. *Nature.* 1990;346(6284):565–7. 10.1038/346565a0 1974037

[40] Paoletti P Ascher P Neyton J: High-affinity zinc inhibition of NMDA NR1-NR2A receptors. *J Neurosci.* 1997;17(15):5711–25. 10.1523/JNEUROSCI.17-15-05711.1997 9221770 PMC6573217

[41] Traynelis SF Burgess MF Zheng F: Control of voltage-independent zinc inhibition of NMDA receptors by the NR1 subunit. *J Neurosci.* 1998;18(16):6163–75. 10.1523/JNEUROSCI.18-16-06163.1998 9698310 PMC6793196

[42] Rachline J Perin-Dureau F Le Goff A: The micromolar zinc-binding domain on the NMDA receptor subunit NR2B. *J Neurosci.* 2005;25(2):308–17. 10.1523/JNEUROSCI.3967-04.2005 15647474 PMC6725474

[43] Groc L Choquet D: AMPA and NMDA glutamate receptor trafficking: Multiple roads for reaching and leaving the synapse. *Cell Tissue Res.* 2006;326(2):423–38. 10.1007/s00441-006-0254-9 16847641

[44] Petralia RS Wang YX Hua F: Organization of NMDA receptors at extrasynaptic locations. *Neuroscience.* 2010;167(1):68–87. 10.1016/j.neuroscience.2010.01.022 20096331 PMC2840201

[45] Sanz-Clemente A Nicoll RA Roche KW: Diversity in NMDA receptor composition: many regulators, many consequences. *Neuroscientist.* 2013;19(1):62–75. 10.1177/1073858411435129 22343826 PMC3567917

[46] Zhou X Ding Q Chen Z: Involvement of the GluN2A and GluN2B subunits in synaptic and extrasynaptic *N*-methyl-D-aspartate receptor function and neuronal excitotoxicity. *J Biol Chem.* 2013;288(33):24151–9. 10.1074/jbc.M113.482000 23839940 PMC3745357

[47] Won S Incontro S Nicoll RA: PSD-95 stabilizes NMDA receptors by inducing the degradation of STEP61. *Proc Natl Acad Sci U S A.* 2016;113(32):E4736-44. 10.1073/pnas.1609702113 27457929 PMC4987792

[48] Delgado JY Fink AE Grant SGN: Rapid homeostatic downregulation of LTP by extrasynaptic GluN2B receptors. *J Neurophysiol.* 2018;120(5):2351–7. 10.1152/jn.00421.2018 30110236 PMC6295522

[49] Hardingham GE Fukunaga Y Bading H: Extrasynaptic NMDARs oppose synaptic NMDARs by triggering CREB shut-off and cell death pathways. *Nat Neurosci.* 2002;5(5):405–14. 10.1038/nn835 11953750

[50] Martel MA Wyllie DJ Hardingham GE: In developing hippocampal neurons, NR2B-containing *N*-methyl-D-aspartate receptors (NMDARs) can mediate signaling to neuronal survival and synaptic potentiation, as well as neuronal death. *Neuroscience.* 2009;158(1):334–43. 10.1016/j.neuroscience.2008.01.080 18378405 PMC2635533

[51] Soriano FX Hardingham GE: Compartmentalized NMDA receptor signalling to survival and death. *J Physiol.* 2007;584(Pt 2):381–7. 10.1113/jphysiol.2007.138875 17690142 PMC2277150

[52] Hardingham GE: Coupling of the NMDA receptor to neuroprotective and neurodestructive events. *Biochem Soc Trans.* 2009;37(Pt 6):1147–60. 10.1042/BST0371147 19909238 PMC2837198

[53] Monyer H Burnashev N Laurie DJ: Developmental and regional expression in the rat brain and functional properties of four NMDA receptors. *Neuron.* 1994;12(3):529–40. 10.1016/0896-6273(94)90210-0 7512349

[54] Hatten ME: Central nervous system neuronal migration. *Annu Rev Neurosci.* 1999;22:511–39. 10.1146/annurev.neuro.22.1.511 10202547

[55] Medina AE Liao DS Mower AF: Do NMDA receptor kinetics regulate the end of critical periods of plasticity? *Neuron.* 2001;32(4):553–5. 10.1016/s0896-6273(01)00514-1 11719195

[56] Dumas TC: Developmental regulation of cognitive abilities: modified composition of a molecular switch turns on associative learning. *Prog Neurobiol.* 2005;76(3):189–211. 10.1016/j.pneurobio.2005.08.002 16181726

[57] Monaco SA Gulchina Y Gao WJ: NR2B subunit in the prefrontal cortex: A double-edged sword for working memory function and psychiatric disorders. *Neurosci Biobehav Rev.* 2015;56:127–38. 10.1016/j.neubiorev.2015.06.022 26143512 PMC4567400

[58] Wyllie DJ Livesey MR Hardingham GE: Influence of GluN2 subunit identity on NMDA receptor function. *Neuropharmacology.* 2013;74:4–17. 10.1016/j.neuropharm.2013.01.016 23376022 PMC3778433

[59] Mori H Mishina M: Structure and function of the NMDA receptor channel. *Neuropharmacology.* 1995;34(10):1219–37. 10.1016/0028-3908(95)00109-j 8570021

[60] Akazawa C Shigemoto R Bessho Y: Differential expression of five N-methyl-D-aspartate receptor subunit mRNAs in the cerebellum of developing and adult rats. *J Comp Neurol.* 1994;347(1):150–60. 10.1002/cne.903470112 7798379

[61] Shi J Townsend M Constantine-Paton M: Activity-dependent induction of tonic calcineurin activity mediates a rapid developmental downregulation of NMDA receptor currents. *Neuron.* 2000;28(1):103–14. 10.1016/s0896-6273(00)00089-1 11086987

[62] van Zundert B Yoshii A Constantine-Paton M: Receptor compartmentalization and trafficking at glutamate synapses: a developmental proposal. *Trends Neurosci.* 2004;27(7):428–37. 10.1016/j.tins.2004.05.010 15219743

[63] McKay S Ryan TJ McQueen J: The Developmental Shift of NMDA Receptor Composition Proceeds Independently of GluN2 Subunit-Specific GluN2 C-Terminal Sequences. *Cell Rep.* 2018;25(4):841–851.e4. 10.1016/j.celrep.2018.09.089 30355491 PMC6218242

[64] Bar-Shira O Maor R Chechik G: Gene Expression Switching of Receptor Subunits in Human Brain Development. *PLoS Comput Biol.* 2015;11(12):e1004559. 10.1371/journal.pcbi.1004559 26636753 PMC4670163

[65] Williams K Russell SL Shen YM: Developmental switch in the expression of NMDA receptors occurs *in vivo* and *in vitro*. *Neuron.* 1993;10(2):267–78. 10.1016/0896-6273(93)90317-k 8439412

[66] Barth AL Malenka RC: NMDAR EPSC kinetics do not regulate the critical period for LTP at thalamocortical synapses. *Nat Neurosci.* 2001;4(3):235–6. 10.1038/85070 11224537

[67] Cohen S Greenberg ME: Communication between the synapse and the nucleus in neuronal development, plasticity, and disease. *Annu Rev Cell Dev Biol.* 2008;24:183–209. 10.1146/annurev.cellbio.24.110707.175235 18616423 PMC2709812

[68] Hall BJ Ripley B Ghosh A: NR2B signaling regulates the development of synaptic AMPA receptor current. *J Neurosci.* 2007;27(49):13446–56. 10.1523/JNEUROSCI.3793-07.2007 18057203 PMC6673095

[69] Kutsuwada T Sakimura K Manabe T: Impairment of suckling response, trigeminal neuronal pattern formation, and hippocampal LTD in NMDA receptor epsilon 2 subunit mutant mice. *Neuron.* 1996;16(2):333–44. 10.1016/s0896-6273(00)80051-3 8789948

[70] Tang YP Shimizu E Dube GR: Genetic enhancement of learning and memory in mice. *Nature.* 1999;401(6748):63–9. 10.1038/43432 10485705

[71] Hu C Chen W Myers SJ: Human *GRIN2B* variants in neurodevelopmental disorders. *J Pharmacol Sci.* 2016;132(2):115–21. 10.1016/j.jphs.2016.10.002 27818011 PMC5125235

[72] XiangWei W Jiang Y Yuan H: *De Novo* Mutations and Rare Variants Occurring in NMDA Receptors. *Curr Opin Physiol.* 2018;2:27–35. 10.1016/j.cophys.2017.12.013 29756080 PMC5945193

[73] Yuan H Hansen KB Zhang J: Functional analysis of a *de novo GRIN2A* missense mutation associated with early-onset epileptic encephalopathy. *Nat Commun.* 2014;5:3251. 10.1038/ncomms4251 24504326 PMC3934797

[74] Lemke JR Geider K Helbig KL: Delineating the *GRIN1* phenotypic spectrum: A distinct genetic NMDA receptor encephalopathy. *Neurology.* 2016;86(23):2171–8. 10.1212/WNL.0000000000002740 27164704 PMC4898312

[75] Endele S Rosenberger G Geider K: Mutations in *GRIN2A* and *GRIN2B* encoding regulatory subunits of NMDA receptors cause variable neurodevelopmental phenotypes. *Nat Genet.* 2010;42(11):1021–6. 10.1038/ng.677 20890276

[76] Reutlinger C Helbig I Gawelczyk B: Deletions in 16p13 including *GRIN2A* in patients with intellectual disability, various dysmorphic features, and seizure disorders of the rolandic region. *Epilepsia.* 2010;51(9):1870–3. 10.1111/j.1528-1167.2010.02555.x 20384727

[77] Burnashev N Szepetowski P: NMDA receptor subunit mutations in neurodevelopmental disorders. *Curr Opin Pharmacol.* 2015;20:73–82. 10.1016/j.coph.2014.11.008 25498981

[78] Yuan H Low CM Moody OA: Ionotropic GABA and Glutamate Receptor Mutations and Human Neurologic Diseases. *Mol Pharmacol.* 2015;88(1):203–17. 10.1124/mol.115.097998 25904555 PMC4468639

[79] Sibarov DA Bruneau N Antonov SM: Functional Properties of Human NMDA Receptors Associated with Epilepsy-Related Mutations of GluN2A Subunit. *Front Cell Neurosci.* 2017;11:155. 10.3389/fncel.2017.00155 28611597 PMC5447064

[80] Fedele L Newcombe J Topf M: Disease-associated missense mutations in GluN2B subunit alter NMDA receptor ligand binding and ion channel properties. *Nat Commun.* 2018;9(1):957. 10.1038/s41467-018-02927-4 29511171 PMC5840332

[81] Traynelis J Silk M Wang Q: Optimizing genomic medicine in epilepsy through a gene-customized approach to missense variant interpretation. *Genome Res.* 2017;27(10):1715–29. 10.1101/gr.226589.117 28864458 PMC5630035

[82] Swanger SA Chen W Wells G: Mechanistic Insight into NMDA Receptor Dysregulation by Rare Variants in the GluN2A and GluN2B Agonist Binding Domains. *Am J Hum Genet.* 2016;99(6):1261–80. 10.1016/j.ajhg.2016.10.002 27839871 PMC5142120

[83] Ogden KK Chen W Swanger SA: Molecular Mechanism of Disease-Associated Mutations in the Pre-M1 Helix of NMDA Receptors and Potential Rescue Pharmacology. *PLoS Genet.* 2017;13(1):e1006536. 10.1371/journal.pgen.1006536 28095420 PMC5240934

[84] Platzer K Yuan H Schütz H: *GRIN2B* encephalopathy: Novel findings on phenotype, variant clustering, functional consequences and treatment aspects. *J Med Genet.* 2017;54(7):460–70. 10.1136/jmedgenet-2016-104509 28377535 PMC5656050

[85] Strehlow V Heyne HO Vlaskamp DR: *GRIN2A*-related disorders: Genotype and functional consequence predict phenotype. *Brain.* 2019;142(1):80–92. 10.1093/brain/awy304 30544257 PMC6308310

[86] Fernández-Marmiesse A Kusumoto H Rekarte S: A novel missense mutation in *GRIN2A* causes a nonepileptic neurodevelopmental disorder. *Mov Disord.* 2018;33(6):992–9. 10.1002/mds.27315 29644724 PMC6105539

[87] Li D Yuan H Ortiz-Gonzalez XR: *GRIN2D* Recurrent *De Novo* Dominant Mutation Causes a Severe Epileptic Encephalopathy Treatable with NMDA Receptor Channel Blockers. *Am J Hum Genet.* 2016;99(4):802–16. 10.1016/j.ajhg.2016.07.013 27616483 PMC5065652

[88] Fry AE Fawcett KA Zelnik N: *De novo* mutations in *GRIN1* cause extensive bilateral polymicrogyria. *Brain.* 2018;141(3):698–712. 10.1093/brain/awx358 29365063 PMC5837214

[89] Pierson TM Yuan H Marsh ED: *GRIN2A* mutation and early-onset epileptic encephalopathy: Personalized therapy with memantine. *Ann Clin Transl Neurol.* 2014;1(3):190–8. 10.1002/acn3.39 24839611 PMC4019449

[90] Chen W Tankovic A Burger PB: Functional Evaluation of a *De Novo GRIN2A* Mutation Identified in a Patient with Profound Global Developmental Delay and Refractory Epilepsy. *Mol Pharmacol.* 2017;91(4):317–30. 10.1124/mol.116.106781 28126851 PMC5363715

[91] Amin JB Leng X Gochman A: A conserved glycine harboring disease-associated mutations permits NMDA receptor slow deactivation and high Ca ^2+^ permeability. *Nat Commun.* 2018;9(4): 3748. 10.1038/s41467-018-06145-w 30217972 PMC6138751

[92] Marwick KF Hansen KB Skehel PA: Functional assessment of triheteromeric NMDA receptors containing a human variant associated with epilepsy. *J Physiol.* 2019;597(6):1691–704. 10.1113/JP277292 30604514 PMC6418762

[93] Xu XX Liu XR Fan CY: Functional Investigation of a *GRIN2A* Variant Associated with Rolandic Epilepsy. *Neurosci Bull.* 2018;34(2):237–46. 10.1007/s12264-017-0182-6 28936771 PMC5856713

[94] Bell S Maussion G Jefri M: Disruption of *GRIN2B* Impairs Differentiation in Human Neurons. *Stem Cell Reports.* 2018;11(1):183–96. 10.1016/j.stemcr.2018.05.018 29937144 PMC6067152

[95] Wells G Yuan H McDaniel MJ: The GluN2B-Glu413Gly NMDA receptor variant arising from a *de novo GRIN2B* mutation promotes ligand-unbinding and domain opening. *Proteins.* 2018;86(12):1265–76. 10.1002/prot.25595 30168177 PMC6774441

[96] Soto D Olivella M Grau C: l-Serine dietary supplementation is associated with clinical improvement of loss-of-function *GRIN2B*-related pediatric encephalopathy. *Sci Signal.* 2019;12(586):eaaw0936. 10.1126/scisignal.aaw0936 31213567

[97] XiangWei W Kannan V Xu Y: Heterogeneous Clinical and Functional Features of *GRIN2D*-related Developmental and Epileptic Encephalopathy. *Brain.* 2019;142(10):3009–3027. 10.1093/brain/awz232 31504254 PMC6763743

[98] Vyklicky V Krausova B Cerny J: Surface Expression, Function, and Pharmacology of Disease-Associated Mutations in the Membrane Domain of the Human GluN2B Subunit. *Front Mol Neurosci.* 2018;11:110. 10.3389/fnmol.2018.00110 29681796 PMC5897658

[99] Carvill GL Regan BM Yendle SC: *GRIN2A* mutations cause epilepsy-aphasia spectrum disorders. *Nat Genet.* 2013;45(9):1073–6. 10.1038/ng.2727 23933818 PMC3868952

[100] Lemke JR Lal D Reinthaler EM: Mutations in *GRIN2A* cause idiopathic focal epilepsy with rolandic spikes. *Nat Genet.* 2013;45(9):1067–72. 10.1038/ng.2728 23933819

[101] Lesca G Rudolf G Bruneau N: *GRIN2A* mutations in acquired epileptic aphasia and related childhood focal epilepsies and encephalopathies with speech and language dysfunction. *Nat Genet.* 2013;45(9):1061–6. 10.1038/ng.2726 23933820

[102] Salmi M Bolbos R Bauer S: Transient microstructural brain anomalies and epileptiform discharges in mice defective for epilepsy and language-related NMDA receptor subunit gene *Grin2a*. *Epilepsia.* 2018;59(10):1919–30. 10.1111/epi.14543 30146685

[103] Lemke JR Hendrickx R Geider K: *GRIN2B* mutations in west syndrome and intellectual disability with focal epilepsy. *Ann Neurol.* 2014;75(1):147–54. 10.1002/ana.24073 24272827 PMC4223934

[104] Kenny EM Cormican P Furlong S: Excess of rare novel loss-of-function variants in synaptic genes in schizophrenia and autism spectrum disorders. *Mol Psychiatry.* 2014;19(8):872–9. 10.1038/mp.2013.127 24126926

[105] O'Roak BJ Deriziotis P Lee C: Exome sequencing in sporadic autism spectrum disorders identifies severe *de novo* mutations. *Nat Genet.* 2011;43(6):585–9. 10.1038/ng.835 21572417 PMC3115696

[106] Adams DR Yuan H Holyoak T: Three rare diseases in one Sib pair: *RAI1, PCK1, GRIN2B* mutations associated with Smith-Magenis Syndrome, cytosolic PEPCK deficiency and NMDA receptor glutamate insensitivity. *Mol Genet Metab.* 2014;113(3):161–70. 10.1016/j.ymgme.2014.04.001 24863970 PMC4219933

[107] de Ligt J Willemsen MH van Bon BW: Diagnostic exome sequencing in persons with severe intellectual disability. *N Engl J Med.* 2012;367(20):1921–9. 10.1056/NEJMoa1206524 23033978

[108] Stessman HA Xiong B Coe BP: Targeted sequencing identifies 91 neurodevelopmental-disorder risk genes with autism and developmental-disability biases. *Nat Genet.* 2017;49(4):515–26. 10.1038/ng.3792 28191889 PMC5374041

[109] von Stülpnagel C Ensslen M Møller RS: Epilepsy in patients with *GRIN2A* alterations: Genetics, neurodevelopment, epileptic phenotype and response to anticonvulsive drugs. *Eur J Paediatr Neurol.* 2017;21(3):530–41. 10.1016/j.ejpn.2017.01.001 28109652

[110] Boutry-Kryza N Labalme A Ville D: Molecular characterization of a cohort of 73 patients with infantile spasms syndrome. *Eur J Med Genet.* 2015;58(2):51–8. 10.1016/j.ejmg.2014.11.007 25497044

[111] Tarabeux J Kebir O Gauthier J: Rare mutations in N-methyl-D-aspartate glutamate receptors in autism spectrum disorders and schizophrenia. *Transl Psychiatry.* 2011;1:e55. 10.1038/tp.2011.52 22833210 PMC3309470

[112] Grozeva D Carss K Spasic-Boskovic O: Targeted Next-Generation Sequencing Analysis of 1,000 Individuals with Intellectual Disability. *Hum Mutat.* 2015;36(12):1197–204. 10.1002/humu.22901 26350204 PMC4833192

[113] Serraz B Grand T Paoletti P: Altered zinc sensitivity of NMDA receptors harboring clinically-relevant mutations. *Neuropharmacology.* 2016;109:196–204. 10.1016/j.neuropharm.2016.06.008 27288002

[114] Retterer K Juusola J Cho MT: Clinical application of whole-exome sequencing across clinical indications. *Genet Med.* 2016;18(7):696–704. 10.1038/gim.2015.148 26633542

[115] Xiong HY Alipanahi B Lee LJ: RNA splicing. The human splicing code reveals new insights into the genetic determinants of disease. *Science.* 2015;347(6218):1254806. 10.1126/science.1254806 25525159 PMC4362528

[116] Addis L Virdee JK Vidler LR: Epilepsy-associated *GRIN2A* mutations reduce NMDA receptor trafficking and agonist potency - molecular profiling and functional rescue. *Sci Rep.* 2017;7(1):66. 10.1038/s41598-017-00115-w 28242877 PMC5427847

[117] DeVries SP Patel AD: Two patients with a *GRIN2A* mutation and childhood-onset epilepsy. *Pediatr Neurol.* 2013;49(6):482–5. 10.1016/j.pediatrneurol.2013.08.023 24125812

[118] Conroy J McGettigan PA McCreary D: Towards the identification of a genetic basis for Landau-Kleffner syndrome. *Epilepsia.* 2014;55(6):858–65. 10.1111/epi.12645 24828792

[119] Møller RS Larsen LH Johannesen KM: Gene Panel Testing in Epileptic Encephalopathies and Familial Epilepsies. *Mol Syndromol.* 2016;7(4):210–9. 10.1159/000448369 27781031 PMC5073625

[120] Farwell KD Shahmirzadi L El-Khechen D: Enhanced utility of family-centered diagnostic exome sequencing with inheritance model-based analysis: results from 500 unselected families with undiagnosed genetic conditions. *Genet Med.* 2015;17(7):578–86. 10.1038/gim.2014.154 25356970

[121] Allen NM Conroy J Shahwan A: Unexplained early onset epileptic encephalopathy: Exome screening and phenotype expansion. *Epilepsia.* 2016;57(1):e12–7. 10.1111/epi.13250 26648591

[122] Zhu X Petrovski S Xie P: Whole-exome sequencing in undiagnosed genetic diseases: interpreting 119 trios. *Genet Med.* 2015;17(10):774–81. 10.1038/gim.2014.191 25590979 PMC4791490

[123] Chen XS Reader RH Hoischen A: Next-generation DNA sequencing identifies novel gene variants and pathways involved in specific language impairment. *Sci Rep.* 2017;7:46105. 10.1038/srep46105 28440294 PMC5404330

[124] Gao K Tankovic A Zhang Y: A *de novo* loss-of-function *GRIN2A* mutation associated with childhood focal epilepsy and acquired epileptic aphasia. *PLoS One.* 2017;12(2):e0170818. 10.1371/journal.pone.0170818 28182669 PMC5300259

[125] Dyment DA Tétreault M Beaulieu CL: Whole-exome sequencing broadens the phenotypic spectrum of rare pediatric epilepsy: a retrospective study. *Clin Genet.* 2015;88(1):34–40. 10.1111/cge.12464 25046240

[126] Gahl WA Mulvihill JJ Toro C: The NIH Undiagnosed Diseases Program and Network: Applications to modern medicine. *Mol Genet Metab.* 2016;117(4):393–400. 10.1016/j.ymgme.2016.01.007 26846157 PMC5560125

[127] Venkateswaran S Myers KA Smith AC: Whole-exome sequencing in an individual with severe global developmental delay and intractable epilepsy identifies a novel, *de novo GRIN2A* mutation. *Epilepsia.* 2014;55(7):e75–9. 10.1111/epi.12663 24903190

[128] Lelieveld SH Reijnders MR Pfundt R: Meta-analysis of 2,104 trios provides support for 10 new genes for intellectual disability. *Nat Neurosci.* 2016;19(9):1194–6. 10.1038/nn.4352 27479843

[129] Butler KM da Silva C Alexander JJ: Diagnostic Yield From 339 Epilepsy Patients Screened on a Clinical Gene Panel. *Pediatr Neurol.* 2017;77:61–6. 10.1016/j.pediatrneurol.2017.09.003 29056246 PMC6885003

[130] Helbig KL Farwell Hagman KD Shinde DN: Diagnostic exome sequencing provides a molecular diagnosis for a significant proportion of patients with epilepsy. *Genet Med.* 2016;18(9):898–905. 10.1038/gim.2015.186 26795593

[131] Bramswig NC Lüdecke HJ Alanay Y: Exome sequencing unravels unexpected differential diagnoses in individuals with the tentative diagnosis of Coffin-Siris and Nicolaides-Baraitser syndromes. *Hum Genet.* 2015;134(6):553–68. 10.1007/s00439-015-1535-8 25724810

[132] Dimassi S Labalme A Lesca G: A subset of genomic alterations detected in rolandic epilepsies contains candidate or known epilepsy genes including *GRIN2A* and *PRRT2*. *Epilepsia.* 2014;55(2):370–8. 10.1111/epi.12502 24372385

[133] Dimassi S Simonet T Labalme A: Comparison of two next-generation sequencing kits for diagnosis of epileptic disorders with a user-friendly tool for displaying gene coverage, DeCovA. *Appl Transl Genom.* 2015;7:19–25. 10.1016/j.atg.2015.10.001 27054081 PMC4803767

[134] Green EK Rees E Walters JT: Copy number variation in bipolar disorder. *Mol Psychiatry.* 2016;21(1):89–93. 10.1038/mp.2014.174 25560756 PMC5038134

[135] O'Roak BJ Vives L Fu W: Multiplex targeted sequencing identifies recurrently mutated genes in autism spectrum disorders. *Science.* 2012;338(6114):1619–22. 10.1126/science.1227764 23160955 PMC3528801

[136] Freunscht I Popp B Blank R: Behavioral phenotype in five individuals with *de novo* mutations within the *GRIN2B* gene. *Behav Brain Funct.* 2013;9:20. 10.1186/1744-9081-9-20 23718928 PMC3685602

[137] Myers RA Casals F Gauthier J: A population genetic approach to mapping neurological disorder genes using deep resequencing. *PLoS Genet.* 2011;7(2):e1001318. 10.1371/journal.pgen.1001318 21383861 PMC3044677

[138] Hildebrand MS Myers CT Carvill GL: A targeted resequencing gene panel for focal epilepsy. *Neurology.* 2016;86(17):1605–12. 10.1212/WNL.0000000000002608 27029629 PMC4844234

[139] Epi4K Consortium; Epilepsy Phenome/Genome Project, Allen AS Berkovic SF: *De novo* mutations in epileptic encephalopathies. *Nature.* 2013;501(7466):217–21. 10.1038/nature12439 23934111 PMC3773011

[140] O'Roak BJ Stessman HA Boyle EA: Recurrent *de novo* mutations implicate novel genes underlying simplex autism risk. *Nat Commun.* 2014;5: 5595. 10.1038/ncomms6595 25418537 PMC4249945

[141] Fokstuen S Makrythanasis P Hammar E: Experience of a multidisciplinary task force with exome sequencing for Mendelian disorders. *Hum Genomics.* 2016;10(1): 24. 10.1186/s40246-016-0080-4 27353043 PMC4924303

[142] Lucariello M Vidal E Vidal S: Whole exome sequencing of Rett syndrome-like patients reveals the mutational diversity of the clinical phenotype. *Hum Genet.* 2016;135(12):1343–54. 10.1007/s00439-016-1721-3 27541642 PMC5065581

[143] Yavarna T Al-Dewik N Al-Mureikhi M: High diagnostic yield of clinical exome sequencing in Middle Eastern patients with Mendelian disorders. *Hum Genet.* 2015;134(9):967–80. 10.1007/s00439-015-1575-0 26077850

[144] Firth HV Richards SM Bevan AP: DECIPHER: *D*atabas *e* of *C*hromosomal *I*mbalance and *P*henotype in *H*umans Using *E*nsembl *R*esources. *Am J Hum Genet.* 2009;84(4):524–33. 10.1016/j.ajhg.2009.03.010 19344873 PMC2667985

[145] Smigiel R Kostrzewa G Kosinska J: Further evidence for *GRIN2B* mutation as the cause of severe epileptic encephalopathy. *Am J Med Genet A.* 2016;170(12):3265–70. 10.1002/ajmg.a.37887 27605359

[146] Bosch DG Boonstra FN de Leeuw N: Novel genetic causes for cerebral visual impairment. *Eur J Hum Genet.* 2016;24(5):660–5. 10.1038/ejhg.2015.186 26350515 PMC4930090

[147] Hamdan FF Srour M Capo-Chichi JM: *De Novo* Mutations in Moderate or Severe Intellectual Disability. *PLoS Genet.* 2014;10(10):e1004772. 10.1371/journal.pgen.1004772 25356899 PMC4214635

[148] Takasaki Y Koide T Wang C: Mutation screening of *GRIN2B* in schizophrenia and autism spectrum disorder in a Japanese population. *Sci Rep.* 2016;6: 33311. 10.1038/srep33311 27616045 PMC5018849

[149] Morisada N Ioroi T Taniguchi-Ikeda M: A 12p13 *GRIN2B* deletion is associated with developmental delay and macrocephaly. *Hum Genome Var.* 2016;3: 16029. 10.1038/hgv.2016.29 27656287 PMC5023786

[150] Dimassi S Andrieux J Labalme A: Interstitial 12p13.1 deletion involving *GRIN2B* in three patients with intellectual disability. *Am J Med Genet A.* 2013;161A(10):2564–9. 10.1002/ajmg.a.36079 23918416

[151] Mishra N Kouzmitcheva E Orsino A: Chromosome 12p Deletion Spanning the GRIN2B Gene Presenting With a Neurodevelopmental Phenotype: A Case Report and Review of Literature. *Child Neurol Open.* 2016;3: 2329048X16629980. 10.1177/2329048X16629980 28503605 PMC5417284

[152] Talkowski ME Rosenfeld JA Blumenthal I: Sequencing chromosomal abnormalities reveals neurodevelopmental loci that confer risk across diagnostic boundaries. *Cell.* 2012;149(3):525–37. 10.1016/j.cell.2012.03.028 22521361 PMC3340505

[153] Griswold AJ Ma D Cukier HN: Evaluation of copy number variations reveals novel candidate genes in autism spectrum disorder-associated pathways. *Hum Mol Genet.* 2012;21(15):3513–23. 10.1093/hmg/dds164 22543975 PMC3392110

[154] Lal D Steinbrücker S Schubert J: Investigation of *GRIN2A* in common epilepsy phenotypes. *Epilepsy Res.* 2015;115:95–9. 10.1016/j.eplepsyres.2015.05.010 26220384

[155] Singh D Lau M Ayers T: *De Novo* Heterogeneous Mutations in SCN2A and GRIN2A Genes and Seizures With Ictal Vocalizations. *Clin Pediatr (Phila).* 2016;55(9):867–70. 10.1177/0009922815601060 26283219

[156] Wollmuth LP Kuner T Sakmann B: Adjacent asparagines in the NR2-subunit of the NMDA receptor channel control the voltage-dependent block by extracellular Mg ^2+^. *J Physiol.* 1998;506(Pt 1):13–32. 10.1111/j.1469-7793.1998.013bx.x 9481670 PMC2230696

[157] Mayer ML: Glutamate receptors at atomic resolution. *Nature.* 2006;440(7083):456–62. 10.1038/nature04709 16554805

[158] Platzer K Lemke JR: *GRIN2B*-Related Neurodevelopmental Disorder.In: Adam MP, Ardinger HH, Pagon RA, *et al.*: GeneReviews® [Internet]. Seattle (WA): University of Washington, Seattle; 1993–2019.2018; May 31. 29851452

[159] Andreoli V de Marco EV Trecroci F: Potential involvement of GRIN2B encoding the NMDA receptor subunit NR2B in the spectrum of Alzheimer's disease. *J Neural Transm (Vienna).* 2014;121(5):533–42. 10.1007/s00702-013-1125-7 24292895

[160] O’Roak BJ Vives L Girirajan S: Sporadic autism exomes reveal a highly interconnected protein network of *de novo* mutations. *Nature.* 2012;485(7397):246–50. 10.1038/nature10989 22495309 PMC3350576

[161] Soto D Altafaj X Sindreu C: Glutamate receptor mutations in psychiatric and neurodevelopmental disorders. *Commun Integr Biol.* 2014;7(1):e27887. 10.4161/cib.27887 24605182 PMC3937208

[162] Delahanty RJ Kang JQ Brune CW: Maternal transmission of a rare GABRB3 signal peptide variant is associated with autism. *Mol Psychiatry.* 2011;16(1):86–96. 10.1038/mp.2009.118 19935738 PMC3428055

[163] Shi YW Zhang Q Cai K: Synaptic clustering differences due to different *GABRB3* mutations cause variable epilepsy syndromes. *Brain.* 2019;142(10):3028–3044. 10.1093/brain/awz250 31435640 PMC6776116

[164] Janve VS Hernandez CC Verdier KM: Epileptic encephalopathy *de novo* GABRB mutations impair γ-aminobutyric acid type A receptor function *Ann Neurol.* 2016;79(5):806–25. 10.1002/ana.24631 26950270 PMC5014730

[165] Lu W Constantine-Paton M: Eye opening rapidly induces synaptic potentiation and refinement. *Neuron.* 2004;43(2):237–49. 10.1016/j.neuron.2004.06.031 15260959

[166] Kotermanski SE Johnson JW: Mg ^2+^ imparts NMDA receptor subtype selectivity to the Alzheimer's drug memantine. *J Neurosci.* 2009;29(9):2774–9. 10.1523/JNEUROSCI.3703-08.2009 19261873 PMC2679254

[167] Glasgow NG Wilcox MR Johnson JW: Effects of Mg ^2+^ on recovery of NMDA receptors from inhibition by memantine and ketamine reveal properties of a second site. *Neuropharmacology.* 2018;137:344–58. 10.1016/j.neuropharm.2018.05.017 29793153 PMC6050087

[168] Perszyk RE DiRaddo JO Strong KL: GluN2D-Containing N-methyl-d-Aspartate Receptors Mediate Synaptic Transmission in Hippocampal Interneurons and Regulate Interneuron Activity. *Mol Pharmacol.* 2016;90(6):689–702. 10.1124/mol.116.105130 27625038 PMC5118640

